# Chronic diseases: Perceptions about Covid-19 risk and vaccination

**DOI:** 10.1101/2021.03.17.21253760

**Published:** 2021-03-24

**Authors:** Emily E. Ricotta, Jennifer L. Kwan, Brianna A. Smith, Nicholas G. Evans

**Affiliations:** Division of Intramural Research, National Institute of Allergy and Infectious Diseases, National Institutes of Health, 5601 Fishers Lane 7D18, Rockville, MD 20852; Division of Intramural Research, National Institute of Allergy and Infectious Diseases; Department of Political Science, United States Naval Academy; Department of Philosophy, University of Massachusetts Lowell

## Abstract

**Background:**

Individuals with chronic disease may be at higher risk of dying from COVID-19, yet no association has been established between chronic illness and COVID-19 risk perception, engagement with nonpharmaceutical interventions (NPIs), or vaccine acceptance.

**Methods:**

We surveyed US residents who self-reported a chronic respiratory or autoimmune disease in February 2021. Respondents reported beliefs about the risk of COVID-19 to personal and public health, adoption and support of NPIs, willingness to be vaccinated against COVID-19, and reasons for vaccination willingness. We evaluated the association between disease status and COVID-19 behaviors or attitudes, adjusting for demographic and political factors.

**Results:**

Compared to healthy controls, chronic disease was associated with increased belief that COVID-19 was a personal (Respiratory = 0.12, 95% confidence interval (CI) = 0.10 – 0.15; Autoimmune = 0.11, CI = 0.08 – 0.14) and public threat (Respiratory = 0.04, CI = 0.02 – 0.06; Autoimmune = 0.03, CI = 0.01 – 0.06), and support for NPIs. Chronic respiratory disease was associated with willingness to be vaccinated (0.6, CI = 0.05 – 0.09). Personal protection was associated with vaccination (Respiratory = 1.08, CI = 1.03 – 1.13; Autoimmune = 1.06, CI = 1.01 – 1.11). Autoimmune disease was associated with fear of a bad vaccine reaction (1.22, CI = 1.06 – 1.41) among those unwilling to be vaccinated.

**Conclusions:**

In the US, chronic disease status is significantly related to risk perceptions of COVID, support of personal and community risk mitigation measures, and willingness to be vaccinated.

## Background

The COVID-19 pandemic disproportionately affects patients with comorbidities,^1–4^ but communication and risk perception around particular patient groups remains understudied. Recent work outside the US has demonstrated that individuals with chronic illnesses and other comorbidities are significantly less likely to refuse to be vaccinated, and are more likely to take personal health-protective measures against COVID-19.^5,6^ Within the US, descriptive statistics show a broad spread of vaccine acceptance for those with underlying medical conditions.^7,8^ However, there is a gap in understanding about key chronic illness patient groups’ risk perceptions of COVID-19, their beliefs about personal and community-level nonpharmaceutical interventions (NPIs), and willingness to be vaccinated.

In the US, acceptance of NPIs and vaccination are critical to ongoing response efforts. In earlier phases of the pandemic, however, concerns were raised about the risk of developing severe COVID-19 for patients with chronic respiratory and autoimmune diseases. The latter’s potential contraindication for vaccine candidates was also of concern, and these patients remain unaddressed in CDC vaccine guidance due to lack of data.^9^ These uncertainties disproportionately affect marginalized groups: non-Hispanic blacks are more likely to experience complications and death from chronic respiratory diseases than non-Hispanic whites,^10^ while women are more likely to have autoimmune diseases than men.^11^ Our study identifies the relationship between an individual’s reported chronic illness, how they perceive the risk of COVID-19, engage with individual and community-level NPIs, and weigh the benefits and risks of vaccination in the US context.

## Methods

Using the Prolific survey platform we recruited US residents who self-reported a chronic respiratory disease, autoimmune disease, both, or neither (“healthy controls”), in February 2021. All respondents were over 18 years of age and US residents. The institutional review board (IRB) of the US Naval Academy approved the study. Informed consent was secured for all participants at the commencement of the survey.

Data were collected on respondent beliefs about the risk of COVID-19 to their health, to the public’s health, and their belief that the risk of COVID-19 is overblown. These risk perceptions were collected on a five-point scale ranging from “strongly agree” to “strongly disagree,” and then normalized to a [0,1] interval where 1 indicates strong agreement with the risk statement. The full survey is available in Supplemental Appendix A.

Adoption of individual risk mitigation measures were collected on a six-point scale ranging from “very false to me” to “very true to me,” normalized to a [0,1] interval where 1 indicates greatest adoption of that measure. Individual risk mitigation measures were reducing trips outside the home; wearing a mask that covers nose and mouth when outside the home; working from home; hand washing more frequently and for a longer amount of time; and maintaining physical distance. Approval of community-level NPIs (limits on indoor dining, mask mandates, limits on in-person worship services, lockdown of non-essential travel, and school closures) were also measured on a six-point scale ranging from “strongly disapprove” to “strongly approve,” normalized to a [0,1] interval where 1 indicates strong approval of that NPI.

Respondents indicated whether they were willing, unwilling, or had already received a COVID-19 vaccine on a seven-point scale, then provided reasons for their vaccine decision. For those willing to be or already vaccinated, reasons included protecting themselves, protecting others, belief the vaccine had been fully tested, belief the vaccine was safe, a desire to get back to normal, or a need to be vaccinated for work or other activities. For those unwilling to get a vaccine, potential reasons included belief that COVID-19 is not a serious health threat, the respondent would have a bad reaction to the vaccine, the vaccine had not been fully tested, the vaccine was not safe, or opposition to vaccines in general. Participants could select multiple reasons, or write their own. Willingness to receive the vaccine was normalized to a [0,1] interval where 1 indicates either that participants would “definitely get the vaccine” or had already been vaccinated.

Demographic questions for age, ethnicity, gender, household income, educational level, geographic region, rural or urban residence, partisanship, employment, and education were collected (Table 1). Respondents were asked if they had been diagnosed with COVID-19 in the past, and whether a family member or close contact had been diagnosed with COVID-19.

We conducted regression analyses to evaluate the association between disease status and COVID-19 behaviors or attitudes using R version 4.0.2,^12^ controlling for the factors listed above. For risk perceptions, individual risk mitigation behaviors, approval of community-level NPIs, and vaccine willingness we conducted OLS regressions. For vaccine attitudes, we conducted logistic regressions. To test whether the slopes of the regression coefficients for “COVID-19 is a threat to me” and “COVID-19 is a threat to the public” were significantly different from one another we used seemingly unrelated regression to account for possible correlation of the equation errors (using the “systemfit” package)^13^ and tested the linear hypothesis using an asymptotic Chi-square test (“car” package).^14^ Results are presented as linear regression coefficients or odds ratios and 95% confidence intervals. Q-values to assess false discovery rate were computed using the Benjamini-Hochburg procedure (Table S1).

## Results

**Figure 1.**
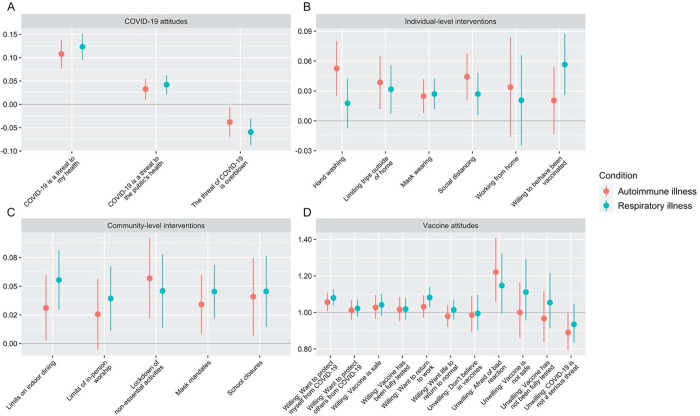
Association between chronic respiratory or autoimmune diseases and public health behaviors. A-C present regression coefficients and 95% confidence intervals; D presents odds ratios and 95% confidence intervals. Horizontal line indicates the null value.

Data were collected from 2535 individuals. Of these, 478 reported having any autoimmune disorder; 618 reported having asthma, chronic obstructive pulmonary disease, or any other chronic respiratory condition; 136 reported having both; and 1303 reported no chronic condition. Regression coefficients and odds ratios can be seen in Figure 1 and Table S1. Outcome variables were normalized to [0,1], so regression coefficients may be interpreted as the percent change in the outcome due to the independent variable.

### Personal beliefs about COVID-19

Compared to healthy controls, respondents reporting chronic respiratory or autoimmune diseases were significantly more likely to report that COVID-19 was a threat to themselves (Respiratory = 0.12, 95% confidence interval (CI) = 0.10 – 0.15; Autoimmune = 0.11, CI = 0.08 – 0.14) and to a lesser extent the public’s health (Respiratory = 0.04, CI = 0.02 – 0.06; Autoimmune = 0.03, CI = 0.01 – 0.06). In both cases, perception of threat to the respondent was significantly higher than to the public (Respiratory χ^2^=21.3, p<0.001; Autoimmune χ^2^=15.2, p<0.001). These individuals were less likely than healthy controls to think the threat of COVID-19 was overblown (Respiratory = −0.06, CI = −0.09 – −0.03; Autoimmune = −0.4, CI = −0.07 – −0.01).

### Acceptance of NPIs

Acceptance of NPIs was broadly concordant with risk perceptions of the threat of COVID: individuals reporting a chronic disease had stronger preferences for NPIs. They were more likely to wear masks outside the home (Respiratory = 0.03, CI = 0.01 – 0.04; Autoimmune = 0.02, CI = 0.01 – 0.04), undertake physical distancing measures (Respiratory = 0.03, CI = 0.01 – 0.05; Autoimmune = 0.04, CI = 0.02 – 0.07), and decrease trips outside the home (Respiratory = 0.03, CI = 0.01 – 0.06; Autoimmune = 0.04, CI = 0.01 – 0.07).

Support for community-level NPIs was higher among individuals who reported chronic respiratory or autoimmune diseases than those without. Both groups were more likely to support prohibitions of indoor dining (Respiratory = 0.06, CI = 0.03 – 0.08; Autoimmune = 0.03, CI = 0.002 – 0.06), broad lockdowns and restrictions on movement (Respiratory = 0.05, CI = 0.01 – 0.08; Autoimmune = 0.06, CI = 0.02 – 0.09), mask mandates (Respiratory = 0.05, CI = 0.02 – 0.07; Autoimmune = 0.03, CI = 0.01 – 0.06), and school closures (Respiratory = 0.05, CI = 0.01 – 0.08; Autoimmune = 0.04, CI = 0.01 – 0.07). The only result that diverged from this pattern was the preference for limits on in-person worship services: while respondents reporting chronic respiratory diseases were significantly more likely than controls to support limits (0.04, CI = 0.01 – 0.07), individuals reporting autoimmune diseases were no more or less likely to prefer those limits than healthy controls (0.03, CI = −0.01 – 0.06).

### Vaccine acceptance

Vaccine acceptance produced more divergent results than NPIs. Respondents who reported chronic respiratory diseases were 5.7% more willing to be vaccinated than healthy respondents (CI = 0.05 – 0.09), while we found no significant difference between individuals with autoimmune diseases and healthy controls (0.02, CI = −0.01 – 0.05). When assessing reasons for being willing or to have been vaccinated, respondents with chronic respiratory disease were 8% more likely (CI = 1.03 – 1.13) and those with autoimmune diseases were 6% more likely (CI = 1.01 – 1.11) to want to be vaccinated to protect themselves from COVID-19. Those who reported having a chronic respiratory disease were also more likely to want to safely return to work (OR = 1.08; CI = 1.03 – 1.14). Among respondents unwilling to be vaccinated, individuals with autoimmune diseases were the only group to have a significant association with a particular cause for hesitancy. These respondents were more likely to report fear of a bad vaccine reaction as the reason for unwillingness to be vaccinated (OR = 1.22; CI = 1.06 – 1.41).

## Discussion

In our analysis of a nationwide survey of individuals with self-reported chronic respiratory or autoimmune conditions, we found these individuals, compared to respondents without a chronic illness, were significantly more likely to be concerned about COVID-19’s threat to the public and, to a significantly greater extent, more concerned about their personal threat from COVID-19. This highlights the significant internalization of risk messaging in these communities and could provide a basis for choosing strategies to communicate public health information based on self or community interests.

Compared to studies evaluating individuals with medically confirmed chronic illnesses,^15^ our respondents were enrolled based on self-identified disease. This, then, provides information about risk perception based on an individual’s beliefs about their disease state, not their diagnosis. Especially for diseases that are difficult to diagnose, such as autoimmune diseases, self-identification with a diagnosis may be a better proxy for risk attitudes (and subsequent uptake of interventions) than medical diagnosis alone. Indeed, respondents with self-reported comorbidities potentially associated with worse COVID-19 clinical outcomes have significantly greater willingness to adopt individual risk management behaviors such as social distancing and mask wearing, and to support a variety of community level interventions. Appealing to individuals based on their self-identified health state may be useful in promoting individual and community public health interventions. For example, one in twelve Americans has asthma,^16^ and framing risk in terms of their comorbidity may generate more support for individual and community level public health measures than more general messaging. A targeted appeal to these individuals may also produce network benefits: given the high prevalence of chronic diseases, most people are likely to know at least one person whose risk perceptions match our findings and may be sympathetic to their concerns.

Vaccine acceptance diverges both in terms of willingness to be vaccinated and specific motivations. Individuals reporting chronic respiratory diseases, but not autoimmune diseases, were more likely to be willing to be vaccinated than controls. While few vaccine uptake studies have focused on individuals with respiratory diseases, at least one study found that individuals with medically confirmed autoimmune diseases were equally willing to be vaccinated as healthy controls.^15^ Among those willing or already vaccinated, both chronic disease groups reported wanting to protect themselves as a motivating factor for vaccination; indeed this is the major result reported among most COVID-19 vaccine attitude studies.^15,17–19^ Additionally, respondents with chronic respiratory diseases, but not autoimmune diseases, were significantly more likely to want to do so because it would help them get back to work. For those unwilling to be vaccinated, respondents with autoimmune diseases were significantly more likely to be vaccine hesitant due to concerns about adverse reactions than controls; this was not the case for individuals who reported chronic respiratory diseases. This relationship may reflect concerns that mRNA vaccines currently dominating the US market may cause higher rates of adverse events in individuals being treated for autoimmune diseases, and has been observed elsewhere.^15,17,20,21^

The heterogeneity between patient populations in key aspects of health behaviors, most significantly in what informs uptake of vaccination, is an important finding for individual and public health communication strategies. These differences may be critical to patient-provider conversations about vaccinations in this and future pandemics. If particular patient populations are more likely to be vaccine hesitant than others, medical specialty groups could focus on patient engagement for vaccine uptake earlier in a pandemic. In the case of COVID-19 and the increased prevalence of safety concerns in individuals with autoimmune diseases who were unwilling to be vaccinated, rheumatologists and other specialists with a higher volume of autoimmune patients are an important resource for helping patients navigate the decision to be vaccinated against pandemic diseases. We did not collect information on whether vaccination motivations were influenced by physicians or other information sources: this will be important for understanding where targeted information about vaccine safety and efficacy should be provided in general, and specifically to vulnerable populations.

This study has some limitations. Adherence to or support for NPIs are self-reported. Social pressure on respondents to report greater affinity toward mask wearing, or support for school closures for example, may be higher given the state of the ongoing pandemic. We note however that the measurement of relative difference between groups was highly significant across a variety of NPIs. It is difficult to imagine why these pressures would differ between groups; further, it is unlikely that social pressures would be greater on individuals reporting chronic diseases than those without to the point that they confound otherwise insignificant results. Additionally, our sample may not be representative of the US public as this study utilized a convenience sample using quotas based on reported disease state. We have controlled for multiple demographic factors, and note that it would be extremely difficult to recruit sufficient populations that satisfied quotas for disease state while maintaining representative sampling. This limitation thus represents a necessary trade-off between our ability to test our key hypotheses about chronic respiratory and autoimmune diseases, and our ability to make statements about the general population. Samples also reflect the real-world social dimensions of these diseases, such as the disproportionate recruitment of women in the autoimmune quota being indicative of the higher prevalence of those diseases among women.

This research provides a timely first look at how important classes of vulnerable patients conceive of COVID-19 risk and adjust their individual behaviors based on their disease status, which has implications for patient care and public health in general. The relationship between disease and acceptance of NPIs can shape how practitioners build support with individuals and communities for ongoing social and personal interventions as vaccine rollout continues. Understanding the relationship between disease and vaccine acceptance allows us to directly address concerns of specific subpopulations as it becomes necessary to vaccinate them and progress to a fully protected population.

## Supplementary Material

Supplement 1

## Figures and Tables

**Table 1.** Characteristics of respondents by reported illness

Variable	Self-reported disease state			
	None (N=1303)	Respiratory (N=618)	Autoimmune (N=478)	Both (N=136)
Age - average years	32.61	35.79	38.49	39.25
Ethnicity - no. (%)				
Non-Hispanic White	872 (67.7)	428 (70.2)	381 (80.4)	108 (79.4)
Black	82 (6.4)	45 (7.38)	22 (4.6)	6 (4.4)
Hispanic or Latino	63 (4.9)	25 (4.1)	22 (4.6)	4 (2.9)
Asian American	180 (14.0)	58 (9.5)	21 (4.4)	7 (5.2)
Native American	6 (0.5)	6 (0.98)	1 (0.2)	2 (1.5)
Two or more Ethnicities	86 (6.7)	48 (7.9)	27 (5.7)	9 (6.6)
Female - no. (%)	673 (51.8)	359 (58.1)	361 (75.5)	111 (81.6)
Education - median	4-year College Degree	2-year College Degree	4-year College Degree	2-year College Degree
Household Income - Median	$60,000–69,999	$50,000–59,999	$50,000–59,999	$40,000–49,999
Region - no. (%)				
West	337 (26.1)	157 (25.6)	105 (22.1)	30 (22.1)
Midwest	258 (20.0)	132 (21.5)	93 (19.5)	32 (23.5)
South	448 (34.7)	199 (32.5)	182 (38.2)	47 (34.6)
Northeast	247 (19.2)	125 (20.4)	96 (20.2)	27 (19.9)
Urban-Rural - mean (0= urban; 1 = rural)	0.42	0.44	0.47	0.47
Partisanship - mean (0 = strong Democrat; 1 = Strong Republican)	0.30	0.26	0.28	0.32
Had COVID themselves - no. (%)	88 (6.8)	39 (6.3)	35 (7.3)	16 (11.8)
Family/close friends had COVID - no. (%)	694 (53.3)	339 (54.9)	297 (62.1)	82 (60.3)
Students - no. (%)	341 (27.1)	157 (25.8)	91 (19.2)	30 (22.4)
Employed full time (%)	569 (48.5)	212 (38.7)	194 (43.5)	23 (19.3)
