# Supplementary material for: Chronic diseases: Perceptions about Covid-19 risk and vaccination
Source: medRxiv. 2021 Mar 24:2021.03.17.21253760. Preprint. [Version 1] doi: 10.1101/2021.03.17.21253760 (PMC8010746; doi:10.1101/2021.03.17.21253760)
Supplement: Supplementary file 1 [file media-1.docx]

# Supplementary Material

**Appendix A: Full survey materials**

COVID-19 is a serious threat to my health.

o Strongly agree  (1)

o Somewhat agree  (2)

o Neither agree nor disagree  (3)

o Somewhat disagree  (4)

o Strongly disagree  (5)

COVID-19 is a serious threat to the health of the public.

o Strongly agree  (1)

o Somewhat agree  (2)

o Neither agree nor disagree  (3)

o Somewhat disagree  (4)

o Strongly disagree  (5)

The threat of COVID-19 is overblown.

o Strongly agree  (1)

o Somewhat agree  (2)

o Neither agree nor disagree  (3)

o Somewhat disagree  (4)

o Strongly disagree  (5)

The coronavirus epidemic has changed some people's lives a lot, and other people's not at all. Please read each of the following statements and rate how true or false they are for you.

You are washing your hands more often and/or for a longer amount of time

o Very false for me  (1)

o Somewhat false for me  (2)

o Slightly false for me  (3)

o Slightly true for me  (4)

o Somewhat true for me  (5)

o Very true for me  (6)

You are working from home

o Very false for me  (1)

o Somewhat false for me  (2)

o Slightly false for me  (3)

o Slightly true for me  (4)

o Somewhat true for me  (5)

o Very true for me  (6)

You keep a distance of six feet between yourself and other people outside your home

o Very false for me  (1)

o Somewhat false for me  (2)

o Slightly false for me  (3)

o Slightly true for me  (4)

o Somewhat true for me  (5)

o Very true for me  (6)

You have reduced your trips outside of home

o Very false for me  (1)

o Somewhat false for me  (2)

o Slightly false for me  (3)

o Slightly true for me  (4)

o Somewhat true for me  (5)

o Very true for me  (6)

You wear a mask or other protection over your nose and mouth when you are outside your home

o Very false for me  (1)

o Somewhat false for me  (2)

o Slightly false for me  (3)

o Slightly true for me  (4)

o Somewhat true for me  (5)

o Very true for me  (6)

How much do you approve of the following ways to limit the spread of the COVID-19 pandemic?

Mandatory mask-wearing in public places

o Strongly approve  (1)

o Somewhat approve  (2)

o Slightly approve (3)

o Slightly disapprove  (4)

o Somewhat disapprove  (5)

o Strongly approve  (6)

Limits on in-person worship or church services

o Strongly approve  (1)

o Somewhat approve  (2)

o Slightly approve (3)

o Slightly disapprove  (4)

o Somewhat disapprove  (5)

o Strongly approve  (6)

Limits on in-door dining

o Strongly approve  (1)

o Somewhat approve  (2)

o Slightly approve (3)

o Slightly disapprove  (4)

o Somewhat disapprove  (5)

o Strongly approve  (6)

Lockdown of all non-essential travel outside the home

o Strongly approve  (1)

o Somewhat approve  (2)

o Slightly approve (3)

o Slightly disapprove  (4)

o Somewhat disapprove  (5)

o Strongly approve  (6)

Closure of schools

o Strongly approve  (1)

o Somewhat approve  (2)

o Slightly approve (3)

o Slightly disapprove  (4)

o Somewhat disapprove  (5)

o Strongly approve  (6)

When a COVID-19 vaccine is made available to you, do you intend to get the vaccine?

o Definitely will get vaccine  (1)

o Probably will get vaccine  (2)

o May get vaccine  (3)

o May not get vaccine  (4)

o Probably will not get vaccine  (5)

o Definitely will not get vaccine  (6)

o I have already received a COVID-19 vaccine  (7)

[IF Definitely will/probably will/may get vaccine OR I have already received a COVID-19 vaccine]

What are the most important factors in your vaccine decision? Select all that apply.

▢ Want to protect myself from getting COVID-19  (1)

▢ Want to protect others from getting COVID-19  (2)

▢ Trust that the vaccine has been fully tested  (3)

▢ Trust that the vaccine is safe  (4)

▢ Want life to go back to normal  (5)

▢ Need the vaccine for work or other activities (6)

▢ Other (7) _____________

[IF Definitely will/probably will/may not get vaccine]

What are the most important factors in your vaccine decision? Select all that apply.

▢ COVID-19 is not a serious health threat (1)

▢ May have a bad reaction to the vaccine (2)

▢ Feel that the vaccine has not been fully tested  (3)

▢ Feel that the vaccine is not safe  (4)

▢ Do not believe in vaccination (5)

▢ Other (6) _____________

Do you identify as having a chronic illness, chronic disease, or disability? Check any that apply.

▢ Chronic illness  (1)

▢ Chronic disease  (2)

▢ Disability  (3)

▢ None of the above  (4)

Do you currently have any of the following chronic health conditions? Check any that apply. Otherwise, just skip this question.

▢ Cancer  (1)

▢ Asthma, Chronic Obstructive Pulmonary Disease (COPD), or any other chronic respiratory condition  (2)

▢ Any auto-immune disorder  (3)

▢ Diabetes  (4)

▢ Any cardiovascular disease  (5)

▢ Other  (6) ________________________________________________

Have you ever tested positive for COVID-19? 

o Yes  (1)

o No  (2)

Do you have any family members or close friends who have ever tested positive for COVID-19?  

o Yes  (1)

o No  (2)

Generally speaking, do you usually think of yourself as a Republican, a Democrat, an Independent, or what?

o Democrat  (1)

o Republican  (2)

o Independent  (3)

o Other party  (4)

[IF Republican or Democrat]

Would you call yourself a strong [Republican/Democrat] or a not very strong [Republican/Democrat]?

o Strong [Republican/Democrat] (1)

o Not very strong [Republican/Democrat] (2)

[IF Independent]

Do you think of yourself as closer to the Republican Party or the Democratic Party?

o Yes, Democratic  (1)

o Yes, Republican  (2)

o No, Neither  (3)

Finally, we would like you to give us a little information about yourself. Please respond to the following background questions.

What is your age? 

[Open numeric entry]

What is your gender?

o Male  (1)

o Female  (2)

o Other  (3) ________________________________________________

Which of the following ethnicities describes you? Select all that apply.

▢ Latino/Hispanic  (1)

▢ Black/African-American  (2)

▢ Asian/Asian-American  (3)

▢ White/Caucasian  (4)

▢ Native American  (5)

▢ Other  (6) ________________________________________________

What is the highest level of education that you have completed?

o Less than High School  (1)

o High School/GED  (2)

o Some College  (3)

o 2-year College Degree  (4)

o 4-year College Degree  (5)

o Master’s Degree  (6)

o Doctoral Degree (Ph.D., J.D., M.D., etc.)  (7)

What is your total family income (including parent income if they list you as a dependent)?

o Less than $10,000  (1)

o $10,000-$19,999  (2)

o $20,000-$29,999  (3)

o $30,000-$39,999  (4)

o $40,000-$49,999  (5)

o $50,000-$59,999  (6)

o $60,000-$69,999  (7)

o $70,000-$79,999  (8)

o $80,000-$89,999  (9)

o $90,000-$99,999  (10)

o $100,000 or greater  (11)

 In which state do you currently reside?

[Drop-down of US States and territories]

Use the scale below to indicate what kind of place you live in most of the time, where 0 is very urban, 2 is suburban, 4 is very rural, and the other numbers are in between. Please note you must move or click the slider to indicate a response.

	[5-point slider scale]

## Table S1—Attitudes about COVID-19 risk and willingness to vaccinate among individuals with autoimmune and respiratory diseases

|  | Respiratory |  |  | Autoimmune |  |  |
| --- | --- | --- | --- | --- | --- | --- |
| Attitudes about COVID-19 |  |  |  |  |  |  |
| Outcome | Coefficient (95% CI*) | p-value | q-value | Coefficient (95% CI) | p-value | q-value |
| COVID-19 is a serious threat to my health | 0.12 (0.10, 0.15) | 0.000 | <0.001 | 0.11 (0.08, 0.14) | <0.001 | <0.001 |
| COVID-19 is a serious threat to the public's health | 0.04 (0.02, 0.06) | 0.000 | <0.001 | 0.03 (0.01, 0.06) | 0.005 | 0.04 |
| The threat of COVID-19 is overblown | -0.06 (-0.09, -0.03) | 0.000 | <0.001 | -0.04 (-0.07, -0.01) | 0.020 | 0.16 |
| Individual-level interventions |  |  |  |  |  |  |
| Outcome | Coefficient (95% CI) | p-value | q-value | Coefficient (95% CI) | p-value | q-value |
| Handwashing | 0.02 (-0.01, 0.04) | 0.17 | 0.68 | 0.05 (0.02, 0.08) | <0.001 | <0.001 |
| Limiting trips outside the home | 0.03 (0.01, 0.06) | 0.01 | 0.09 | 0.04 (0.01, 0.07) | 0.005 | 0.06 |
| Mask wearing | 0.03 (0.01, 0.04) | 0.001 | 0.02 | 0.02 (0.01, 0.04) | 0.004 | 0.03 |
| Social distancing | 0.03 (0.01, 0.05) | 0.01 | 0.17 | 0.04 (0.02, 0.07) | <0.001 | <0.001 |
| Working from home | 0.02 (-0.02, 0.07) | 0.38 | 0.77 | 0.03 (-0.02, 0.08) | 0.19 | 0.83 |
| Willingness to receive a COVID-19 vaccine* | 0.06 (0.03, 0.09) | <0.001 | <0.001 | 0.02 (-0.01, 0.05) | 0.24 | 0.85 |
| Community-level interventions |  |  |  |  |  |  |
| Outcome | Coefficient (95% CI) | p-value | q-value | Coefficient (95% CI) | p-value | q-value |
| Limits on indoor dining | 0.06 (0.03, 0.08) | <0.001 | <0.001 | 0.03 (0.00, 0.06) | 0.03 | 0.29 |
| Limits on in-person worship services | 0.04 (0.01, 0.07) | 0.006 | 0.06 | 0.03 (-0.01, 0.06) | 0.11 | 0.81 |
| Lockdown of all non-essential travel | 0.05 (0.01, 0.08) | 0.005 | 0.04 | 0.06 (0.02, 0.09) | 0.002 | 0.03 |
| Mandatory mask-wearing in public | 0.05 (0.02, 0.07) | 0.000 | <0.001 | 0.03 (0.01, 0.06) | 0.01 | 0.11 |
| School closures | 0.05 (0.01, 0.08) | 0.004 | 0.03 | 0.04 (0.01, 0.07) | 0.02 | 0.14 |
| Vaccine attitudes |  |  |  |  |  |  |
| Outcome | Odds ratio (95% CI) | p-value | q-value | Odds ratio (95% CI) | p-value | q-value |
| Willing: Want to protect myself from COVID-19 | 1.08 (1.03, 1.13) | <0.001 | <0.001 | 1.06 (1.01, 1.11) | 0.02 | 0.20 |
| Willing: Want to protect others from COVID-19 | 1.02 (0.97, 1.07) | 0.37 | 0.74 | 1.01 (0.96, 1.07) | 0.66 | 1.00 |
| Willing: Vaccine is safe | 1.04 (0.98, 1.10) | 0.16 | 0.57 | 1.03 (0.96, 1.10) | 0.41 | 0.89 |
| Willing: Vaccine has been fully tested | 1.02 (0.96, 1.08) | 0.55 | 0.92 | 1.02 (0.95, 1.08) | 0.63 | 0.96 |
| Willing: Want to return to work | 1.08 (1.03, 1.14) | 0.003 | 0.03 | 1.03 (0.97, 1.09) | 0.31 | 0.88 |
| Willing: Want life to return to normal | 1.01 (0.96, 1.07) | 0.62 | 0.92 | 0.98 (0.92, 1.04) | 0.50 | 0.92 |
| Unwilling: Don't believe in vaccines | 0.99 (0.90, 1.10) | 0.92 | 0.92 | 0.99 (0.89, 1.09) | 0.77 | 1.00 |
| Unwilling: Afraid of bad reaction | 1.15 (1.00, 1.32) | 0.06 | 0.47 | 1.22 (1.06, 1.41) | 0.006 | 0.09 |
| Unwilling: Vaccine is not safe | 1.11 (0.96, 1.29) | 0.17 | 0.71 | 1.00 (0.86, 1.16) | 1.00 | 1.00 |
| Unwilling: Vaccine has not been fully tested | 1.05 (0.91, 1.22) | 0.48 | 0.82 | 0.97 (0.84, 1.12) | 0.64 | 1.00 |
| Unwilling: COVID-19 is not a serious threat | 0.93 (0.84, 1.05) | 0.24 | 0.72 | 0.89 (0.80, 1.00) | 0.04 | 0.53 |
| *Inclusive of individuals already vaccinated. A separate regression excluding vaccinated respondents did not change the results. |  |  |  |  |  |  |
